# A comparative study of one-step vs. two-step approach in the treatment of pediatric gallbladder stones with common bile duct stones

**DOI:** 10.3389/fped.2026.1802412

**Published:** 2026-04-08

**Authors:** Junjun Ju, Tao Zhang, Xueyi Hu, Hualong Chen, Chenxi Ding, Shiqin Qi, Zhubin Pan

**Affiliations:** Department of General Surgery, Anhui Provincial Children's Hospital, Hefei, Anhui, China

**Keywords:** anesthesia, cholecystectomy, common bile duct stones, endoscopic retrograde, endoscopic retrograde cholangiopancreatography (ERCP), gallbladder stones, pediatrics

## Abstract

**Objective:**

This study aims to compare the effects of the one-step and two-step methods of ERCP combined with laparoscopic cholecystectomy in the clinical application of pediatric gallbladder stones with common bile duct stones.

**Methods:**

Clinical data were retrospectively collected from pediatric patients diagnosed with gallbladder stones combined with common bile duct stones at our hospital from January 2023 to January 2026. Patients were divided into two groups based on the surgical approach: the one-step group (underwent ERCP and laparoscopic cholecystectomy under the same anesthesia) and the two-step group (underwent ERCP first, followed by laparoscopic cholecystectomy in a separate session). Baseline data and perioperative indicators were compared between the two groups.

**Results:**

A total of 76 pediatric patients were included in the study, with 29 patients in the one-step group and 47 patients in the two-step group. There were no significant differences in the preoperative baseline data (gender, age, BMI, common bile duct diameter, and preoperative liver function indicators) between the two groups (all *P* > 0.05). The total anesthesia time in the one-step group [320.0 (270.1, 337.5) mins] was significantly shorter than that of the two-step group [361.0 (324.4, 369.7) mins] (Z = −2.314, *P* = 0.019), and the length of hospitalization in the one-step group (5.3 ± 1.2) days was significantly shorter than the two-step group (6.8 ± 1.4) days (t = 4.784, *P* < 0.001). There were no significant differences between the two groups in total surgery time [273.2 (190.5, 320.4) mins vs. 287.3 (200.4, 347.1) mins, *P* = 0.9], postoperative stone clearance rate (89.6% vs. 87.2%, *P* = 0.249), postoperative complications (bile leakage, pancreatitis, cholangitis, etc.), postoperative liver function indicators (total bilirubin, direct bilirubin, AST, ALT), and hospitalization costs (all *P* > 0.05).

**Conclusion:**

The one-step ERCP combined with laparoscopic cholecystectomy is safe and feasible in the treatment of pediatric gallbladder stones with common bile duct stones. The one-step method reduces the number of anesthesia sessions and anesthesia time, lowers the risk of general anesthesia in children, and shortens the length of hospitalization, making it worthy of clinical promotion in pediatrics.

## 1 Introduction

Gallbladder stones combined with common bile duct stones, though still relatively rare in children, have shown an increasing incidence in recent years. Approximately 0.13%–1.9% of children are diagnosed with gallbladder stones during ultrasound examinations, and the incidence is closely related to risk factors such as increasing age, obesity, genetics, and hemolytic diseases (1). Stone migration or bile stasis can both lead to the formation of bile duct stones. Among patients with gallbladder stones, it is estimated that 4%–20% also have common bile duct stones, according to adult literature. In pediatric patients, the incidence of common bile duct stones is higher than in adults, with up to 30% of children presenting with biliary stones during preoperative examinations (2).

The treatment involves using ERCP to clear the stones from the common bile duct, followed by laparoscopic cholecystectomy to remove the gallbladder filled with multiple stones. The traditional treatment approach is divided into two steps: first performing ERCP to clear the common bile duct stones, followed by a scheduled laparoscopic cholecystectomy. However, this method involves two anesthesia processes, which increase the anesthesia risk and hospitalization time for the child.

To reduce the number of anesthesia sessions while minimizing the risk of complications, some scholars have proposed combining ERCP and laparoscopic cholecystectomy under a single anesthesia, known as the one-step method (3). The advantage of the one-step method is that it reduces the number of anesthesia sessions and lowers anesthesia risks. However, the feasibility and safety of the one-step method remain a focus of clinical attention, with limited pediatric experience.

This study aims to compare the clinical effects of the one-step method and the traditional two-step method in the treatment of pediatric gallbladder stones combined with common bile duct stones. It evaluates the differences in anesthesia time, hospitalization time, postoperative complication rates, and provides a theoretical basis for clinical standardized treatment.

## 2 Methods

### 2.1 General information

**Figure 1:**
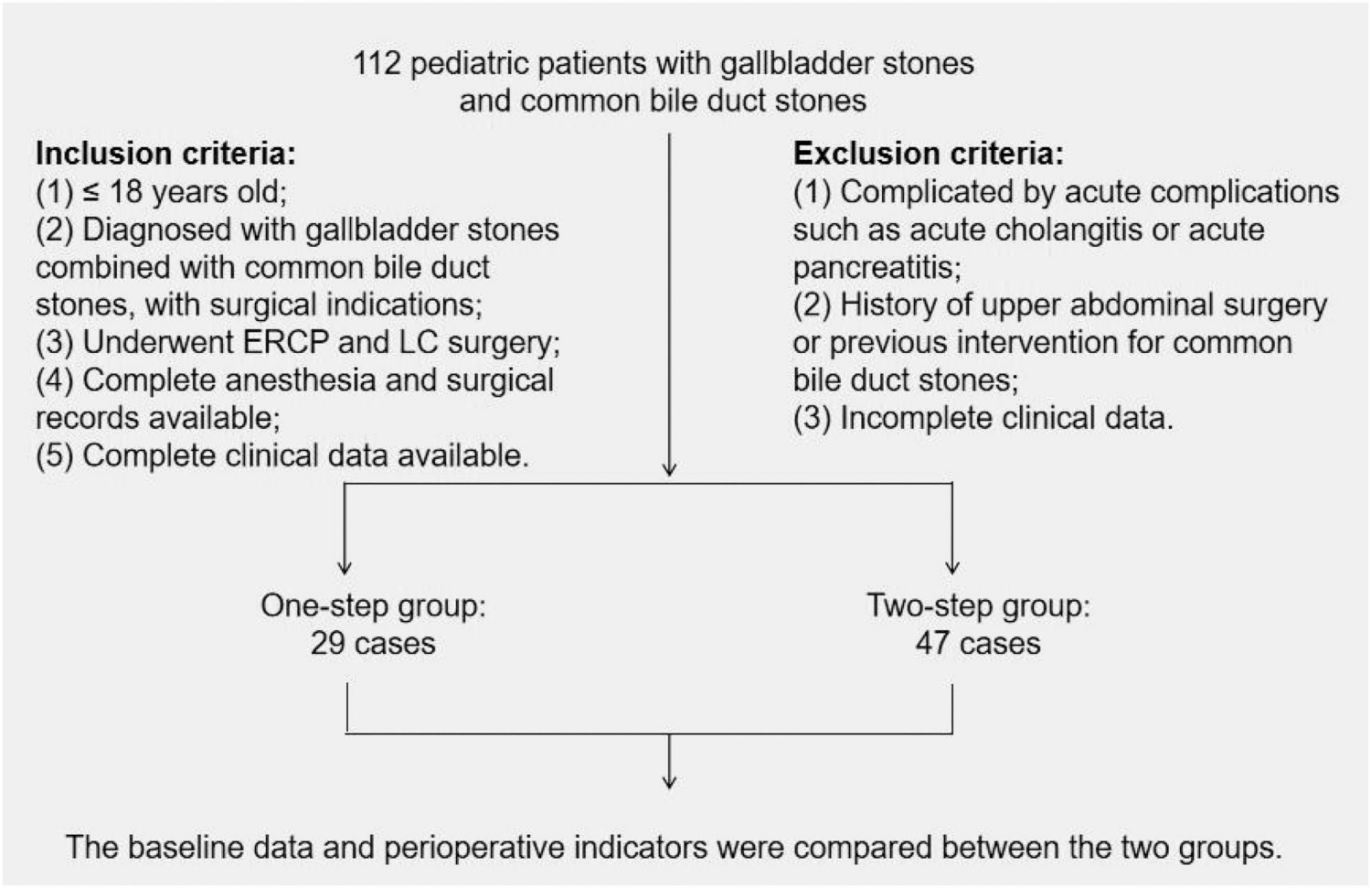
Case selection flowchart of the study.

This study is a retrospective case-control study. Clinical data were collected from pediatric patients diagnosed with gallbladder stones combined with common bile duct stones at our institution from January 2023 to January 2026. All children were diagnosed through preoperative imaging examinations and met the surgical indications. Patients were divided into two groups based on the surgical approach: the one-step group (underwent ERCP and laparoscopic cholecystectomy under the same anesthesia) and the two-step group (underwent ERCP first, followed by scheduled laparoscopic cholecystectomy). This study was approved by the hospital's medical ethics committee, and informed consent was obtained from all patients and their families. The case selection flowchart for this study is shown in Figure 1.

### 2.2 Inclusion and exclusion criteria

#### 2.2.1 Inclusion criteria

(1) Age of the child ≤18 years; (2) Initial diagnosis of gallbladder stones combined with common bile duct stones based on clinical symptoms, laboratory tests, and imaging examinations, with preoperative MRCP confirming the presence of common bile duct stones and clear surgical indications; (3) Patients who received ERCP and LC at our hospital, not suitable for stone extraction with gallbladder preservation, or whose families requested gallbladder removal, divided into the one-step or two-step groups; (4) No severe organ dysfunction, no contraindications for ERCP or LC surgery, and able to tolerate anesthesia and surgery; (5) Complete clinical data, with signed informed consent from the child and their family.

#### 2.2.2 Exclusion criteria

(1) Presence of acute complications such as acute cholangitis or acute pancreatitis; (2) History of upper abdominal surgery or previous intervention for common bile duct stones; (3) Common bile duct stones with a diameter >1.5 cm that cannot be removed through ERCP; (4) Severe coagulation disorders or other serious diseases that cannot tolerate surgery; (5) Incomplete clinical data or lost to follow-up.

### 2.3 Study indicators

The main clinical data collected include: (1) Baseline data: gender, age, body mass index (BMI), preoperative common bile duct diameter, and preoperative liver function indicators (aspartate aminotransferase, alanine aminotransferase, total bilirubin, direct bilirubin); (2) Surgery and anesthesia-related indicators: total anesthesia time, total surgery time; (3) Hospitalization-related indicators: length of hospitalization, hospitalization costs; (4) Postoperative outcome indicators: stone clearance rate, postoperative complications (including pancreatitis, bile leakage, infection, bleeding, etc.).

Primary Endpoints: Total anesthesia time, length of hospitalization.

Secondary Endpoints: Stone clearance rate, postoperative complication rate, postoperative liver function markers, hospitalization costs.

Total Anesthesia Time: Defined as the total duration from the start of anesthesia induction to the end of recovery. For the two-step procedure group, total anesthesia time is the sum of the anesthesia times for ERCP during the first hospitalization and for LC during the second hospitalization. Data were obtained from the original anesthesia records of both surgeries.

Length of Hospitalization: Defined as the total number of days from admission to discharge. For the two-step procedure group, this is the sum of the hospitalization days for both stays, with the actual discharge date recorded in the medical order form being used as the reference.

Hospitalization Costs: Defined as the sum of the costs for both hospitalizations, based on the hospitalization cost records in the case files of each hospitalization.

Stone clearance rate was defined as the proportion of children in whom no residual stones were confirmed in the common bile duct and gallbladder fossa by imaging examinations after all scheduled surgeries. Evaluation criteria: All children underwent routine abdominal ultrasonography within 72 h postoperatively (before discharge) as the initial assessment. Two senior radiologists reviewed the images independently in a blinded manner, focusing on the acoustic shadow of common bile duct stones and intrahepatic bile duct dilatation. If ultrasonography was unclear due to intestinal gas interference, the imaging findings were suspicious, or postoperative clinical indicators (e.g., insignificant decrease in liver function, persistent abdominal pain) highly suggested possible residual stones, magnetic resonance cholangiopancreatography (MRCP) was added for confirmatory evaluation, with images reviewed jointly by radiologists and surgeons. Complete stone clearance was defined as the absence of definite acoustic shadow of stones on ultrasonography and no intraductal filling defect on the supplementary MRCP. For the two-step group, the assessment was uniformly performed before discharge from the second hospitalization (after laparoscopic cholecystectomy, LC).

Post-ERCP Pancreatitis (PEP) Definition: PEP is defined as meeting at least two of the following three criteria: 1. New or worsening abdominal pain post-surgery; 2. Serum amylase and/or lipase levels greater than three times the upper normal limit; 3. Imaging findings consistent with acute pancreatitis. Prophylactic measures for children at high risk of PEP include preoperative rectal administration of nonsteroidal anti-inflammatory drugs (indomethacin suppositories), preferential use of balloon dilation for stone extraction during the procedure, and placement of a prophylactic pancreatic stent.

### 2.4 Surgical steps

All surgeries were performed by a pediatric surgery team to ensure consistency and standardization in the procedures. Preoperative assessments included routine blood biochemistry, liver function, coagulation function, and serum amylase/lipase tests. The imaging evaluation primarily involved magnetic resonance cholangiopancreatography (MRCP), supplemented by abdominal ultrasound to clarify stone characteristics and biliary anatomy. All patients received endotracheal intubation general anesthesia.

#### 2.4.1 One-step procedure group (simultaneous ERCP+laparoscopic cholecystectomy)

ERCP Stone Extraction: The patient was placed in the left lateral decubitus position, and an adult duodenoscope was used to insert through the mouth into the descending duodenum. A pediatric-specific sphincterotome and guidewire were placed, followed by selective biliary cannulation and cholangiography. Papillary treatment prioritized balloon dilation, and sphincterotomy was performed for impacted stones or poor dilation response. Stone extraction was performed using balloon extraction; for hard stones, mechanical lithotripsy with a basket was attempted. Post-procedure cholangiography was conducted to confirm stone removal, and a nasobiliary drainage tube or biliary stent was placed as needed. No conversions to open surgery were recorded.

Laparoscopic Cholecystectomy (LC): After ERCP, the patient was immediately repositioned to the supine position. A Triport single-port technique was used with an umbilical incision, and CO₂ pneumoperitoneum was established (pressure was adjusted based on age, between 6 and 12 mmHg). Pediatric-specific laparoscopic instruments were used to explore the abdomen, free the cystic duct and artery, and ligate them using absorbable clips. The gallbladder was dissected with an ultrasonic scalpel, and hemostasis was achieved. The gallbladder was extracted through the umbilical incision. After confirming no bleeding or bile leakage, the incision was closed.

#### 2.4.2 Two-step procedure group (staged ERCP+laparoscopic cholecystectomy)

First Stage (ERCP Stone Extraction): The procedural steps were the same as in the one-step procedure. The ERCP procedure involved balloon dilation or sphincterotomy for papillary management, with stent placement and mechanical lithotripsy used when appropriate. There were no conversions to open surgery. After the procedure, the patient was transferred to a general ward for monitoring of vital signs and drainage fluid, with follow-up blood tests for serum amylase, lipase, and liver function. Patients were discharged once the criteria were met.

Second Stage (LC): This was performed 2–6 weeks after ERCP, once inflammation had subsided and liver function had returned to normal. The procedure followed the same steps as in the one-step LC group, with meticulous attention given to adhesions.

### 2.5 Statistical methods

SPSS 27.0 statistical software was used to analyze all clinical data in this study. First, the Kolmogorov–Smirnov test was used to assess the normality of quantitative variables: For normally distributed data with equal variance [such as age, BMI, preoperative common bile duct diameter, postoperative liver function indicators (ALT, AST, total bilirubin, direct bilirubin), total anesthesia time, total surgery time, length of hospitalization, hospitalization costs, etc.], the data are expressed as (mean ± standard deviation), and independent samples t-test was used for comparisons between the two groups. For non-normally distributed quantitative data, data are expressed as M(Q1, Q3), with Mann–Whitney U test used for comparisons between two groups and Kruskal–Wallis test for multiple group comparisons. Categorical variables (such as gender, postoperative complication rates, stone clearance rate, etc.) are expressed as frequency (%), with *χ*2 test used for group comparisons. If a 2 × 2 table has a sample size *n* < 40 or if any cell in the table has a frequency T < 1, Fisher's exact test is applied. A *P* value of <0.05 was considered statistically significant.

## 3 Results

### 3.1 Baseline characteristics

A total of 76 pediatric patients were included in the study, with 29 patients in the one-step group and 47 patients in the two-step group. The case selection flowchart for this study is shown in Figure 1. The preoperative baseline data of the two groups of children were comparable. There were no statistical differences between the groups in terms of gender, age, BMI, common bile duct diameter, and preoperative liver function indicators (total bilirubin, direct bilirubin, AST, ALT) (all *P* > 0.05), as shown in Table 1.

**Table 1:** Comparison of preoperative baseline data between the two groups of children.

Parameter	One-step group (*N* = 29)	Two-step group (*N* = 47)	*χ2/T/Z* value	*P* value
Sex (male/female, *n*)	11/18	17/30	*0.025*	*0.875*
Age (year, $\bar{x}\pm s$)	11.13 ± 3.55	12.28 ± 2.94	*1.529*	*0.130*
BMI (kg/m^2^, $\bar{x}\pm s$)	19.30 ± 2.50	19.70 ± 2.60	*0.661*	*0.510*
diameter of the CBD (mm, $\bar{x}\pm s$)	8.51 ± 2.20	9.24 ± 2.32	*1.310*	*0.195*
Tbil (μmol/ L, $\bar{x}\pm s$)	85.47 ± 26.83	83.28 ± 19.98	*0.407*	*0.685*
Dbil [μmol/ L, M(Q1, Q3)]	41.8 (32.2, 52.9)	40.0 (30.1, 50.2)	*0.372*	*0.710*
AST [U/ L, M(Q1, Q3)]	268.5 (255.3, 275.1)	257.5 (251.4, 280.9)	*1.943*	*0.052*
ALT [U/ L, M(Q1, Q3)]	310.8 (308.4, 335.2)	296.3 (290.4, 339.1)	*1.672*	*0.095*

Italic values indicate statistical test values (*χ², T*, or *Z*) and *P* values.

*P* < 0.05 indicates that the difference is statistically significant. CBD, common bile duct; Tbil, total bilirubin; Dbil, direct bilirubin; AST, aspartate aminotransferase; ALT, alanine aminotransferase.

### 3.2 Anesthesia and surgical indicators

In the comparison between the two groups, the total anesthesia time was significantly longer in the two-step group than in the one-step group (*P* = 0.019), and the length of hospitalization in the two-step group was significantly longer than in the one-step group (*P* < 0.001). However, there were no statistical differences between the two groups in total surgery time, postoperative liver function indicators (total bilirubin, direct bilirubin, AST, ALT), or hospitalization costs (*P* > 0.05), as shown in Table 2.

**Table 2:** Comparison of anesthesia and surgical outcomes between the two groups of children.

Parameter	One-step group (*N* = 29)	Two-step group (*N* = 47)	*χ2/T/Z* value	*P* value
Total anesthesia time [mins, M(Q1, Q3)]	320.0 (270.1,337.5)	361.0 (324.4,369.7)	*−2.314*	0.019*
Total operation time [mins, M(Q1, Q3)]	273.2 (190.5,320.4)	287.3 (200.4,347.1)	*−0.125*	*0.900*
Tbil [μmol/ L, M(Q1, Q3)]	36.72 (33.14,46.24)	37.72 (34.64,47.61)	*−0.094*	*0.925*
Dbil [μmol/ L, M(Q1,Q3)]	19.17 (15.32,24.26)	19.25 (15.52,26.75)	*−0.010*	*0.992*
AST [U/ L, M(Q1, Q3)]	119.6 (114.3, 128.4)	120.5 (113.9, 127.4)	*−0.080*	*0.936*
ALT [U/ L, M(Q1, Q3)]	168.3 (155.2, 178.1)	173.3 (167.8, 197.4)	*−0.231*	*0.817*
Length of hospital stay (day, $\bar{x}\pm s$)	5.3 ± 1.2	6.8 ± 1.4	*4.784*	<0.001*
Hospitalization cost (RMB, $\bar{x}\pm s$)	17,890.4 ± 2,376.4	18,752 ± 3,012.4	*1.384*	*0.195*

Values in italics represent the corresponding test statistic (*χ²* value*, t* value, or *Z* value).

*Indicates that the difference is statistically significant (*P* < 0.05).

### 3.3 Postoperative complication rate

There was no significant difference in the postoperative stone clearance rate between the one-step group and the two-step group (*P* = 0.249). Regarding postoperative complications, there were no statistical differences between the one-step group and the two-step group in terms of the incidence of bile leakage, pancreatitis, cholangitis, bleeding, or incision infection (*P* > 0.05), as shown in Table 3. In this study, all PEP cases were mild, and the patients recovered after conservative treatment, including fasting, aggressive fluid resuscitation, and analgesia. The hospitalization time was extended by 2–3 days, with no moderate or severe cases.

**Table 3:** Comparison of postoperative stone clearance rate and postoperative complications between the two groups of children.

Parameter	One-step group (*N* = 29)	Two-step group (*N* = 47)	*χ2/T/Z* value	*P* value
Postoperative stone clearance, *n* (%)	22 (75.9%)	37 (78.7%)	0.085	0.771
Postoperative complications, *n* (%)				
Bile leakage	1 (3.4%)	2 (4.2%)	0.028	0.867
Pancreatitis	4 (13.8%)	3 (6.4%)	1.190	0.275
Cholangitis	1 (3.4%)	1 (2.1%)	0.028	0.867
Hemorrhage	0 (0.0%)	0 (0.0%)	-	1.000
Incision infection	1 (3.4%)	0 (0.0%)	-	1.000

*P* < 0.05 indicates that the difference is statistically significant.

### 3.4 Follow-up results

Both groups of patients completed a 6-month postoperative follow-up. During the follow-up period, there were no cases of stone recurrence or readmission in the one-step procedure group. In the two-step procedure group, one patient (2.1%) required a second ERCP for stone extraction 3 months post-surgery due to residual stones (a missed diagnosis of cystic duct remnant stones on preoperative MRCP). The patient recovered and did not experience any recurrence. No delayed complications (such as biliary strictures, recurrent cholangitis, or recurrent pancreatitis) have been observed in either group. Liver function returned to normal ranges in both groups upon the 6-month postoperative review.

## 4 Discussion

This study retrospectively analyzed the clinical data of 76 pediatric patients with gallbladder stones combined with common bile duct stones, comparing the treatment effects of ERCP combined with laparoscopic cholecystectomy (LC) using the one-step method and the two-step method. The results showed that there were no statistical differences between the two groups in terms of surgical safety (postoperative complication rate, stone clearance rate), surgical efficiency (total surgery time), and postoperative liver function recovery (all *P* > 0.05). However, the one-step method had significant advantages in reducing the number of anesthesia sessions, shortening hospitalization time, and controlling hospitalization costs (*P* < 0.05), and the total anesthesia time was significantly shorter than the two-step method (*P* = 0.019). These results are consistent with several recent studies on pediatric common bile duct stone treatment, further confirming the safety and feasibility of the one-step method in pediatric clinical applications, providing important clinical evidence for optimizing the treatment strategy for pediatric gallbladder stones combined with common bile duct stones (4–6).

Although the incidence of pediatric gallbladder stones combined with common bile duct stones is lower than that in adults, it has been increasing in recent years. Given that children's physiological functions are not yet fully developed, controlling the risks of anesthesia and surgery is particularly critical (7). The traditional two-step method involves a staged treatment approach with ERCP and LC, requiring two general anesthesia sessions. This not only increases the number of anesthesia exposures but may also extend the hospitalization period due to repeated biliary inflammation, stone displacement, and other issues between the two surgeries (8, 9). In this study, the total anesthesia time in the two-step group was significantly longer at 361.0 (324.4, 369.7) mins compared to the one-step group at 320.0 (270.1, 337.5) mins, and the length of hospitalization (6.8 ± 1.4) days was significantly longer in the two-step group than the one-step group (5.3 ± 1.2) days. This aligns closely with Sanin et al.'s (10) research conclusion on the “surgery-first” strategy, which can shorten hospitalization time and reduce anesthesia exposure. The one-step method, by completing ERCP stone removal and LC under the same anesthesia, effectively avoids the potential risks associated with two anesthesia sessions (such as anesthetic drug accumulation and respiratory and circulatory suppression), while also preventing fluctuations in the patient's condition between staged surgeries. This significantly improves treatment efficiency, which is of critical clinical significance for pediatric patients.

The question of whether the one-step or two-step procedure is superior remains controversial. The one-step procedure involves a single, prolonged anesthesia session, while the two-step procedure involves two separate, shorter anesthesia sessions, with the cumulative risks of both approaches needing to be assessed in pediatric patients from the perspectives of both “duration” and “frequency” of anesthesia. A 2024 study published in Anesthesiology showed that children who underwent multiple anesthesia sessions before the age of 5 had an average IQ score that was 5.8 points lower than those who had single or no anesthesia, suggesting that an increased number of anesthesia sessions may have a cumulative effect on neurodevelopment (11). However, a 2025 meta-analysis found that anesthesia lasting more than 3 h was associated with an increased risk of attention deficit hyperactivity disorder and learning disabilities (HRs of 1.99 and 1.71, respectively), indicating that prolonged single anesthesia sessions also pose potential risks (12). Additionally, intraoperative patient transfer itself can lead to hemodynamic fluctuations and other adverse events, which should be mitigated through optimized procedural protocols. In summary, there is no definitive conclusion regarding the risk balance between the two approaches: the one-step procedure reduces the number of anesthesia sessions but extends the duration and carries transfer risks, while the two-step procedure distributes the total duration but increases the number of anesthesia sessions. This study found no significant differences in short-term outcomes between the two groups, but long-term neurodevelopmental outcomes will require further follow-up. Therefore, the choice of procedure should be based on a comprehensive assessment of the child's age, underlying conditions, and the surgical team's ability to collaborate. The conclusions of this study are applicable only to carefully selected, stable pediatric patients and should be conducted at centers with mature collaborative processes.

Surgical safety and effectiveness are core considerations in pediatric surgical treatment. The results of this study show that there were no statistical differences between the two groups in postoperative stone clearance rates (one-step method 89.6% vs. two-step method 87.2%, *P* = 0.249) or the incidence of complications (such as bile leakage, pancreatitis, cholangitis, etc.) (all *P* > 0.05), suggesting that the one-step method can achieve comparable clinical outcomes to the two-step method without compromising treatment effectiveness or increasing the risk of complications. This finding aligns with Guo et al.'s (13) view that selecting an appropriate surgical strategy can ensure treatment safety while avoiding unnecessary endoscopic procedures. It is noteworthy that in this study, the incidence of postoperative pancreatitis was low in both groups (one-step method 13.8%, two-step method 6.4%), with no severe complications, thanks to the standardized surgical procedures. For example, in ERCP, balloon dilation was prioritized to preserve sphincter function and avoid cannula misplacement into the pancreatic duct, and in LC, low-power ultrasound was used for precise operation. These meticulous techniques effectively reduced the risk of postoperative complications and further validated the rationality and safety of the one-step method's procedural flow.

From a health economics and healthcare resource utilization perspective, the advantages of the one-step method are also significant. In this study, the hospitalization costs in the one-step group were lower than those in the two-step group, although the difference did not reach statistical significance (*P* = 0.171), and the reduction in hospitalization time could lower the cost of family caregiving and indirect economic losses while decreasing hospital bed occupancy, thus optimizing healthcare resource allocation (14, 15). The traditional two-step method, due to its staged surgeries, requires multiple preoperative evaluations and postoperative monitoring, which not only increases medical expenses but may also reduce treatment adherence due to concerns from patients and families about undergoing multiple surgeries. In contrast, the one-step method optimizes the process, controlling medical costs while ensuring treatment quality, in line with the current trends in precision and efficient healthcare, especially suitable for pediatric settings with relatively limited medical resources.

Although this study has yielded meaningful conclusions, there are certain limitations. First, this is a single-center retrospective case-control study with a relatively small sample size. No multivariate analysis or propensity score correction was performed to account for potential confounding factors, and there is a degree of selection bias (for example, the choice of surgical method may be influenced by factors such as the severity of the child's condition and family preferences). Second, the study did not include long-term follow-up of the children, so the long-term outcomes of the one-step method, such as stone recurrence and biliary function recovery, remain unclear. Long-term prognostic evaluation is crucial for formulating treatment strategies for pediatric chronic diseases. Additionally, the study did not analyze in detail the impact of stone size and quantity on the efficacy of the two surgical methods, while previous studies have shown that stone characteristics are important factors affecting the treatment outcome of common bile duct stones (16–18).

Based on the results of this study and existing literature evidence, the one-step method could be considered as the preferred treatment strategy for pediatric gallbladder stones combined with common bile duct stones in future clinical practice, especially for children with stones ≤1.5 cm in diameter, no acute complications, and the ability to tolerate a single long anesthesia session. Additionally, further prospective multi-center studies should be conducted to expand the sample size and long-term follow-up to assess the long-term efficacy of the one-step method. Furthermore, individualized treatment plans based on stone characteristics (size, number, location), as well as the child's age and physiological condition, could be explored to enable more precise treatment decisions.

For pediatric patients with stable conditions and no acute complications, the one-step ERCP combined with laparoscopic cholecystectomy demonstrates advantages in terms of shorter anesthesia exposure time, shorter hospitalization duration, and manageable medical costs when treating children with gallbladder stones and concomitant common bile duct stones. Its safety and efficacy are comparable to the traditional two-step procedure. It is important to note that this study is a single-center, small-sample retrospective analysis, and it excluded patients with concomitant acute cholangitis or pancreatitis. Therefore, the conclusions are only applicable to carefully selected elective surgical cases. In centers with pediatric endoscopy and surgical collaboration teams, this approach shows preliminary feasibility for eligible patients. However, its broader applicability in the general population requires further validation through large-sample, prospective studies. Overall, the one-step procedure aligns with the pediatric surgical principles of “precision, efficiency, and safety”. Under strict adherence to the indications, it can be considered as one of the standardized treatment options for pediatric patients with gallbladder stones and concomitant common bile duct stones, and it is worth promoting in eligible patients.

## Data Availability

The original contributions presented in the study are included in the article/Supplementary Material, further inquiries can be directed to the corresponding authors.
